# Author Correction: Evolution of the potassium channel gene *Kcnj13* underlies colour pattern diversification in *Danio* fish

**DOI:** 10.1038/s41467-021-23570-6

**Published:** 2021-05-20

**Authors:** Marco Podobnik, Hans Georg Frohnhöfer, Christopher M. Dooley, Anastasia Eskova, Christiane Nüsslein-Volhard, Uwe Irion

**Affiliations:** 1 Max Planck Institute for Developmental Biology, Max-Planck-Ring 5, 72076 Tübingen, Germany; 2 Present Address: Max Planck Institute for Heart and Lung Research, Ludwigstrasse 43, 61231 Bad Nauheim, Germany; 3 Present Address: IBM Research and Development, Schönaicher Straße 220, 71032 Böblingen, Germany

**Keywords:** Evolutionary genetics, Evolutionary biology

Correction to: *Nature Communications* 10.1038/s41467-020-20021-6, published online 4 December 2020.

The original version of this Article contained an error in Fig. 4, in which Fig. 4h was inadvertently duplicated and included in Fig 4f also. The correct version of panels Fig. 4e–h is 

which replaces the previous incorrect version:

This has been corrected in both the PDF and HTML versions of the Article.

