# Supplementary material for: Evolution of the potassium channel gene Kcnj13 underlies colour pattern diversification in Danio fish
Source: Nat Commun. 2020 Dec 4;11:6230. doi: 10.1038/s41467-020-20021-6 (PMC7718271; doi:10.1038/s41467-020-20021-6)
Supplement: Supplementary file 1 — Supplementary Information [file 41467_2020_20021_MOESM1_ESM.pdf]

Supplementary Information

Evolution of the potassium channel gene *Kcnj13* underlies colour pattern diversification in *Danio* fish

Marco Podobnik, Hans Georg Frohnhöfer, Christopher M. Dooley, Anastasia Eskova, Christiane Nüsslein-Volhard, Uwe Irion

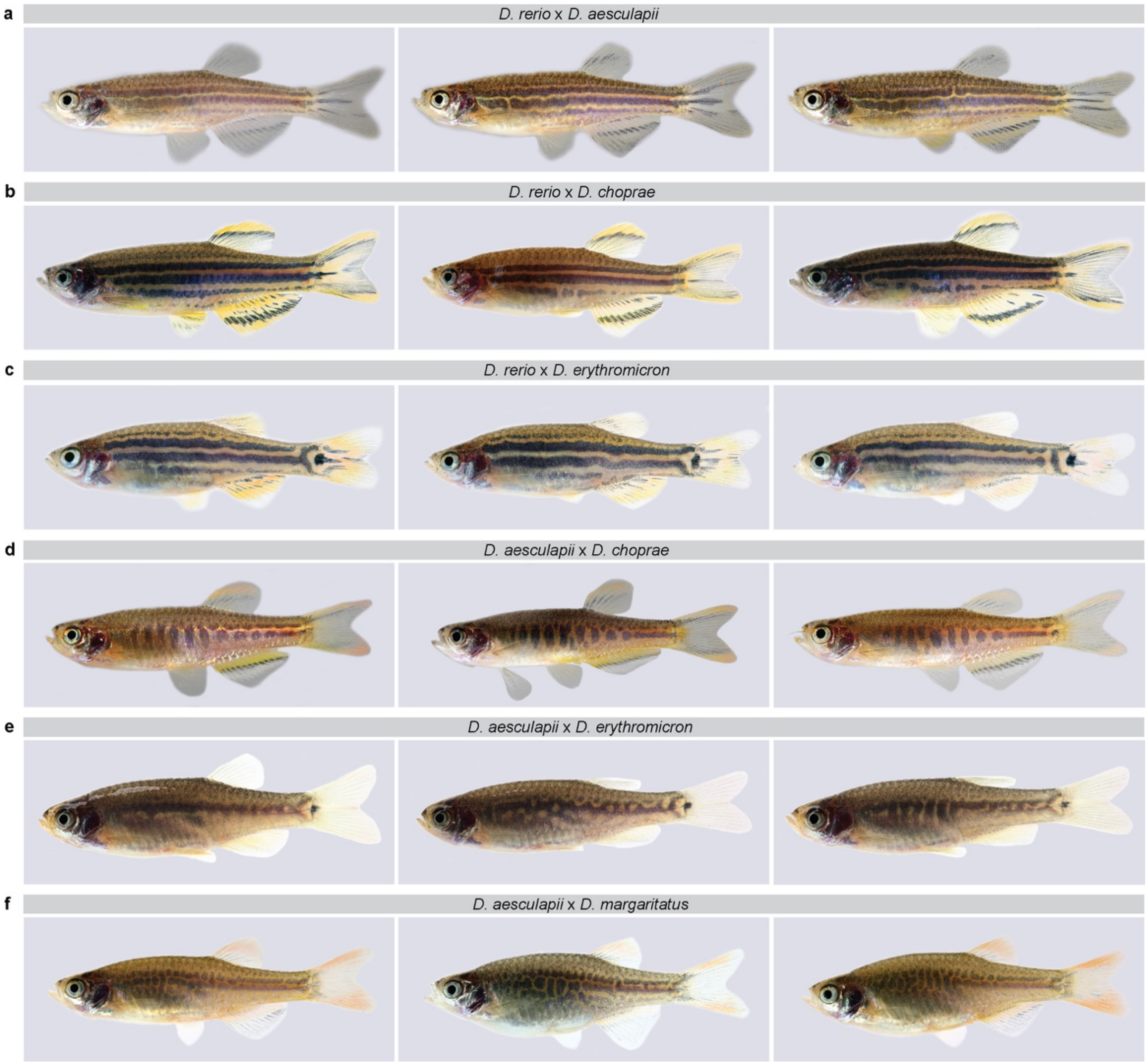

Supplementary Fig. 1: Examples of wild-type hybrids.

In each row three different individuals of wild-type hybrids are shown. **a**, Hybrids between *D. rerio* and *D. aesculapii*, **b**, *D. rerio* and *D. choprae* and **c**, *D. rerio* and *D. erythromicron* all develop horizontal stripes similar to the *D. rerio* pattern. **d**, Hybrids between *D. aesculapii* and *D. choprae* develop a barred pattern similar to the parental species. **e**, Hybrids between *D. aesculapii* and *D. erythromicron* and **f**, hybrids between *D. aesculapii* and *D. margaritatus* show variable patterns without clear horizontal or vertical orientation.

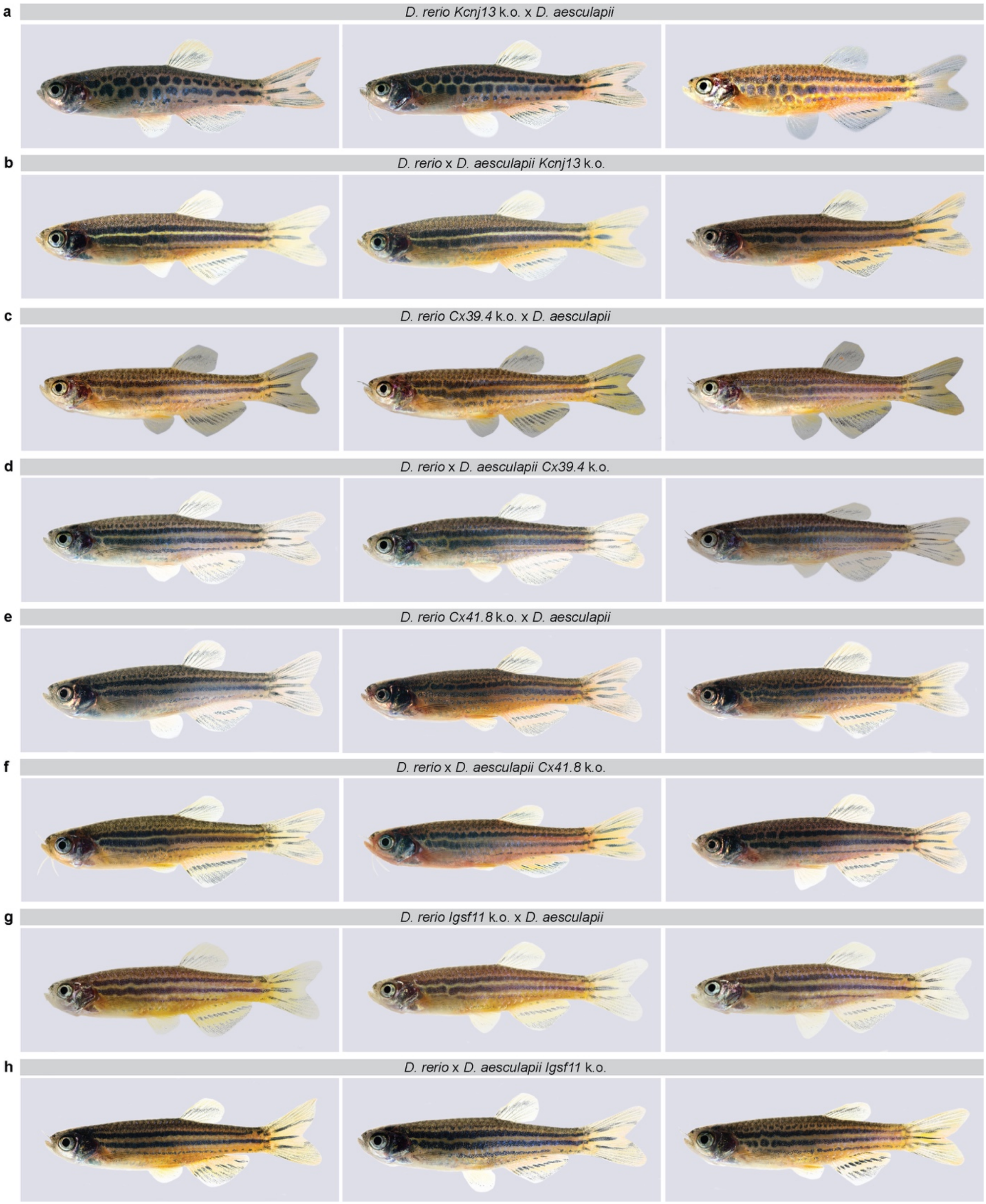
a
D. rerio Kcnj13 k.o. x D. aesculapii
b
D. rerio x D. aesculapii Kcnj13 k.o.
c
D. rerio Cx39.4 k.o. x D. aesculapii
d
D. rerio x D. aesculapii Cx39.4 k.o.
e
D. rerio Cx41.8 k.o. x D. aesculapii
f
D. rerio x D. aesculapii Cx41.8 k.o.
g
D. rerio Igsf11 k.o. x D. aesculapii
h
D. rerio x D. aesculapii Igsf11 k.o.

Supplementary Fig. 2: Examples of hemizygous hybrids.

In each row three different individuals of hemizygous hybrids between *D. rerio* and *D. aesculapii* are shown. **a**, Only the hybrids carrying a mutant *Kcnj13* allele from *D. rerio* show a spotted pattern, different from wild-type hybrids. **b**, Hybrids with the mutant *Kcnj13* allele from *D. aesculapii* and, **c**/**d**, all hemizygous hybrids for *Cx39.4*, **e**/**f**, *Cx41.8*, and, **g**/**h**, *Igsf11* develop horizontal stripes indistinguishable from wild-type hybrids.

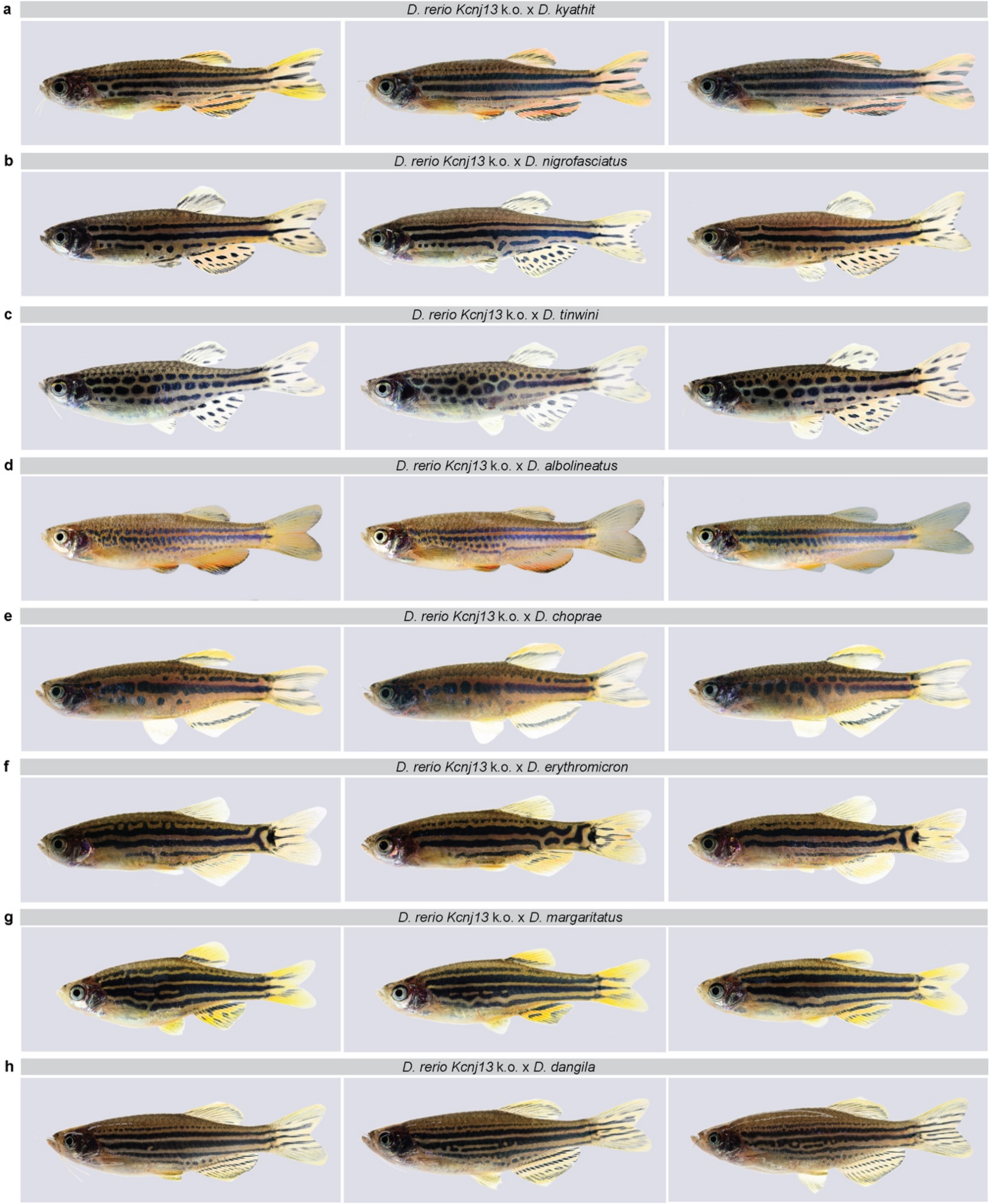
a
D. rerio Kcnj13 k.o. x D. kyathit
b
D. rerio Kcnj13 k.o. x D. nigrofasciatus
c
D. rerio Kcnj13 k.o. x D. tinwini
d
D. rerio Kcnj13 k.o. x D. albolineatus
e
D. rerio Kcnj13 k.o. x D. choprae
f
D. rerio Kcnj13 k.o. x D. erythromicron
g
D. rerio Kcnj13 k.o. x D. margaritatus
h
D. rerio Kcnj13 k.o. x D. dangila

Supplementary Fig. 3: Examples for *Danio* hybrids with mutant *Kcnj13* from *D. rerio*.

In each row three different individuals of hybrids carrying the mutant *Kcnj13* allele from *D. rerio* are shown. **a**, Hybrids with *D. kyathit*, **b**, *D. nigrofasciatus*, **d**, *D. albolineatus*, **f**, *D. erythromicron*, **g**, *D. margaritatus*, **h**, and *D. dangila* are indistinguishable from wild-type hybrids, whereas hybrids with, **c**, *D. tinwini* and, **e**, *D. choprae* show spotted patterns different from the corresponding wild-type hybrids.

```
                                                     A  L   S/N                                                 dΔΔV/G
D. rerio          1 MP----TTMTNTTAD-QKASCPLMVKPQRRRLVSKDGRSQTRGNTRGGSRETCFSALRDLWGTWLALRWRWVVLA  70
L. oculatus       1 MA----AK--R-NED-RKASTPLIAL-RRRRLVTKDGHCTLRNTSHASAPAKGLLYLQDIWSTLVDLRWRWVLLA  69
X. tropicalis     1 MVLQIRTDRDE-DTNSRSPKCTLLSP-CTHRLVTKDGHSMVKAG---RMQGQGLTYLRDIWGLLLDMRWRWMMLA  70
A. carolinensis   1 MR----TEVIE-GNN-TKPTAPLLTQ-RYPRMVTKDGHSTLQID---GAQGKGLAYLKDAWGILMDMRWRWMMLV  65
G. fortis         1 MT----TDTLE-SNN-TKSSAPLLSQ-RYLRLVTKDGHSTFHMA---GAQGTGVSYLRDVWGILMDMRWRWMMLV  65
G. gallus         1 MR----TDTTE-SNN-TKSSTPLLTQ-RYLRMVTKDGHSTFQMD---GAQGQGLAYLRDAWGILMDMRWRWMMLV  59
M. musculus       1 M---------D-SSN-CKVNAPLLSQ-RHRRMVTKDGHSTLQMD---GAQ-RGLVYLRDAWGILMDMRWRWMMLV  59
H. sapiens        1 M---------D-SSN-CKVIAPLLSQ-RYRRMVTKDGHSTLQMD---GAQ-RGLAYLRDAWGILMDMRWRWMMLV  59
cons.               *              :  .   .*:      *:*:***:.  :           .  *:* *.  : :****::*.
                                                                                                rW→X

                                                                                       dL→F
                                                                                     dT→M
                             M1                                                    H5
D. rerio         71 FCGSFLLHWLLFAVLWYLLARVNGDLDVLDHDSPPPGHVLCVKHVNGFTAAFSFALETQLTIGYGTMYPNADCPT 145
L. oculatus      70 FCISFLAHWLLFACLWYLLAHLNGDLG-VDHDNPPPGHTVCVKYITSFTAAFSFSLETQLTIGYGTMFPSGDCPS 144
X. tropicalis    71 FSASFLAHWLLFAVFWYLLAEMNGDLA-VDHDAPPENHTICVKYITSFTAAFSFSLETQLTIGYGTMFPSGDCPS 145
A. carolinensis  66 FSASFVLHWLVFAVLWYLLAEMNGDLE-IDHDSPPENHTICVKYITSFTAAFSFSLETQLTIGYGTMFPSGDCPS 140
G. fortis        66 FSASFLLHWLVFAVLWYLLADMNGDLE-LDHDAPPDNHTICVKYVTSFTAAFSFSLETQLTIGYGTMFPSGDCPS 140
G. gallus        60 FSASFVIHWLVFAVLWYVLAEMNGDLE-LDHDSPPDNHTICVKYITSFTAAFSFSLETQLTIGYGTMFPSGDCPS 134
M. musculus      60 FSASFVVHWLVFAVLWYAVAEMNGDLE-IDHDVPPENHTICVKHITSFTAAFSFSLETQLTIGYGTMFPSGDCPS 134
H. sapiens       60 FSASFVVHWLVFAVLWYVLAEMNGDLE-LDHDAPPENHTICVKYITSFTAAFSFSLETQLTIGYGTMFPSGDCPS 134
cons.               *. **: ***:** :** :* :****  :*** ** .*.:***:..********:************:*..***:
                                                                  dS→I           rI→T

                            dG→E                        G                R/H      L/P
                  rA→X          M2        dF→L          G              G/S      N/T    T      A/S
D. rerio        146 AIALLALQMLLGLMLEAFITGAFVAKFSRPQKRCDGILFSPQAVVCEQKGQRCLMFRVCNLQPQPLVDVSVSAVL 220
L. oculatus     145 AIALLAVQMLLGLMLEAIITGAFVAKIARPKKRAGTIKFSSSAVVGHHQGETCLMFRAANMRDSPLTEVRVSAIL 218
X. tropicalis   146 AIALLAVQMLLGLMLEAFITGVFVAKIARPKNRTPSIRFSRLAVVGSPEGKPCLMFQVANTRSSPLTMVKVSGIL 219
A. carolinensis 141 AIALLAIQMVLGLMLEAFITGAFVAKIARPKNRALSIRFTYSAVVTHREGKPYLMFQVANTRPSPLTSVRISAVL 214
G. fortis       141 AIALLAIQMLLGLMLEAFLTGAFVAKIARPKNRAHSIRFSRSAVVTHSEGKPQLMFQVANTRSSPLTNVQISAIL 214
G. gallus       135 AIALLAIQMVLGLMLEAFITGAFVAKIARPKNRAFSIRFTRSAVVTHTEGKPYLMFQVANTRSSPLTSVQISAIL 208
M. musculus     135 AIALLAIQMLLGLMLEAFITGAFVAKIARPKNRAFSIRFTDLAVVAHKDGKPNLIFQVANTRPSPLTNVRVSAVL 208
H. sapiens      135 AIALLAIQMLLGLMLEAFITGAFVAKIARPKNRAFSIRFTDTAVVAHMDGKPNLIFQVANTRPSPLTSVRVSAVL 208
cons.               ******:**:*******::**.****::**::*    * *:   ***     .*:   *:*:..* : .**. * :*.:*
                                          rT→I      dR→WrR→X

                                E                     K
D. rerio        221 YEERDDHELHQTALEFSIDNL-GSRSCPLFLSPLTFFHPLNPSTPFIN--NPS---SQTHFELVVFLTATQESTG 289
L. oculatus     219 YQEREDQILHQTSVEFHLDRL-RGPECPFFIFPLTFYHPLTEYSPLYSVLCEG---NPAHFELVVFLSATQEGTG 289
X. tropicalis   220 YQERGDGQIQQANVEFSLDSQASGAECPFFTFPLTYSHSLCSGSPLSVLLQRELLSSAGHLELVVCLSATQESSG 294
A. carolinensis 215 YEEQENGQLHQTSVDFHLDSI-TSEECPFFIFPLTYYHSISPSSPLAILLQRE---AHRHFELVVFLSATQEGTG 285
G. fortis       215 YQEQESGQLHQTSIDFHLDSI-TAHEYPFFIFPLTYYHTITASSPLAALLQRE---APPHFELVVFLSAVQEGTG 285
G. gallus       209 YQEQENGQLHQTSIDFHLDSI-TLDECPFFTFPLTYYHSIIPPSPLAALLQRE---AAHHFELVVFLSAVQEGTG 279
M. musculus     209 YQERENGELYQTSVDFHLDGI-SSEECPFFIFPLTYYHTISPSSPLATLLQHE---TPPHFELVVFLSAMQEGTG 279
H. sapiens      209 YQERENGKLYQTSVDFHLDGI-SSDECPFFIFPLTYYHSITPSSPLATLLQHE---NPSHFELVVFLSAMQEGTG 279
cons.               *:*:  .  : *: ::* :*       . *:*  ***: *.:    :*:             *:**** *:* **.:*
                          rQ→X                 rL→P

                                               T/M R        dY→X V/A Q
D. rerio        290 SGYHKRTSYLPDEIQYGYCFSKVTSVHQN--KTPNMRYFDTVPCPLTLANTHTTDTPDKEHVVVQLNGEGSDRV- 361
L. oculatus     290 EGCQKRTSYVREEIQVDYHFASVLGLDPHGSYKVNTTNLNKVLP--DPSHT-TLDGEK--VFVIQINGDGNDGIG 359
X. tropicalis   295 EICQCRTSYLPSEILQGHRFAPCLTRRLEGGYRICMESFGRPLP--ELPQP-SHRQLYRTDLEVCANGQRGDTFQ 365
A. carolinensis 286 ETCQRRTSYLPSEIILHHHFASMLVRGAKGEYQIKMENFDKTIP--ELPGA-DPKSSKRTDMEIRINGQHMDSFQ 357
G. fortis       286 ETCQRRTSYLSSEILVYHRFASLLGHNAKGEYETKMENFDKTVP--EFPAALDLKSPKRTDKEIRINGQHIDSFQ 358
G. gallus       280 EICQRRTSYLPSEIMLYHRFAPVLARSAKGEYQIKMENFDKTIP--ELPAAADSMNPMRTAKEIRINGQHVDSFQ 332
M. musculus     280 EICQRRTSYLPSEIMLHHRFAALMTRGSKGEYQVKMENFDKTVP--EHPTPVVSKSPHRTDLDIHINGQSIDNFQ 332
H. sapiens      280 EICQRRTSYLPSEIMLHHCFASLLTRGSKGEYQIKMENFDKTVP--EFPTPLVSKSPNRTDLDIHINGQSIDNFQ 332
cons.                .  : ****: .**    : *:                    :.  :      . .              :   **:   * .

D. rerio        361 -------E  362
L. oculatus     359 -------G  360
X. tropicalis   366 ICETGLGE  374
A. carolinensis 358 ICETRLTE  365
G. fortis       359 LSETGLTK  366
G. gallus       333 LSETGLTE  360
M. musculus     333 IAETGLTE  360
H. sapiens      333 ISETGLTE  360
cons.
```

Supplementary Fig. 4: Kcnj13 sequence alignment of vertebrate orthologues.

Sequence alignment of Kcnj13 orthologues from different vertebrate species. The two transmembrane domains (M1/M2) are shaded in light grey, the P-Loop (H5) in dark grey. Dominant (d) or recessive (r) mutations are indicated for zebrafish[1-5] (blue) and human[6-12] (purple). Positions that are different between *D. rerio* and *D. aesculapii* (magenta), *D. tinwini* (yellow), *D. choprae* (cyan) and polymorphic positions in *D. rerio* (dark grey) are highlighted. Kcnj13 sequences of *Danio rerio* (zebrafish, NP_001039014.1), *Lepisosteus oculatus* (spotted gar, XP_006638004.1), *Xenopus tropicalis* (tropical clawed frog, NP_001096437.1), *Anolis carolinensis* (green anole, XP_016847621.1), *Geospiza fortis* (medium ground finch, XP_005430275.1), *Gallus gallus* (chicken, XP_015132697.1), *Mus musculus* (house mouse, NP_001103697.1) and *Homo sapiens* (human, NP_002233.2).

```
                                                            S/N                             dΔΔV/G
D. rerio            1 MPTTMTNTTADQKASCPLMVKPQRRRLVSKDGRSQTRGNTRGGSRETCFSALRDLWGTWLALRWRWVVLAFCGSF  75
D. aesculapii       1 MPTTMTNTTADQKASCPLMVKPLRRRLVSKDGRSQTRGNTRGGSRETCFSALRDLWGTWLALRWRWVVLAFCGSF  75
D. kyathit          1 MPTTMTNTTADQKASCPLMVKPKRRRLVSKDGRSQTRGNTRGGSRETCFSALRDLWGTWLALRWRWVVLAFCGSF  75
D. nigrofasciatus   1 MPTTMTNTTADQKASCPLMVKPKRRRLVSKDGRSQTRGNTRGGSRETCFSALRDLWGTWLALRWRWVVLAFCGSF  75
D. tinwini          1 MPTTMTNTTADQKASCPLMVKPQRRRLVSKDGRSQTRGNTRGGSRETCFSALRDLWGTWLALRWRWVVLAFCGSF  75
D. albolineatus     1 MPTTMTNTTADQKASCPLMVKPQRRRLVSKDGRSQTRGNTRGGSRETCFSALRDLWGTWLALRWRWVVLAFCGSF  75
D. choprae          1 MPTTMTNTTADQKASCPLMAKPQRRRLVSKDGRSQTRGNTRGGSRETCFSALRDLWGTWLALRWRWVVLAFCGSF  75
D. erythromicron    1 MPTTMTNTPADQKASCPLMVKPQRRRLVSKDGRSRTRGSTRGGSRETCFSALRDLWGTWLALRWRWVVLAFCGSF  75
D. margaritatus     1 MPTTMTNTTADQKASCPLMVKPQRRRLVSKDGRSRTRGSTRGGSRETCFSALRDLWGTWLALRWRWVVLAFCGSF  75
cons.                 ********.**********.** ***********:***.************************************

                                                                           dL→F
                                                                         dT→M
                             M1                                           H5               rA→X
D. rerio           76 LLHWLLFAVLWYLLARVNGDLDVLDHDSPPPGHVLCVKHVNGFTAAFSFALETQLTIGYGTMYPNADCPTAIALL 150
D. aesculapii      76 LLHWLLFAVLWYLLARVNGDLDVLDHDSPPPGHVLCVKHVNGFTAAFSFALETQLTIGYGTMYPNADCPTAIALL 150
D. kyathit         76 LLHWLLFAVLWYLLARVNGDLDVLDHDSPPPGHVLCVKHVNGFTAAFSFALETQLTIGYGTMYPNADCPTAIALL 150
D. nigrofasciatus  76 LLHWLLFAVLWYLLARVNGDLDVLDHDSPPPGHVLCVKHVNGFTAAFSFALETQLTIGYGTMYPNADCPTAIALL 150
D. tinwini         76 LLHWLLFAVLWYLLARVNGDLDVLDHDSPPPGHVLCVKHVNGFTAAFSFALETQLTIGYGTMYPNADCPTAIALL 150
D. albolineatus    76 LLHWLLFAVLWYLLARVNGDLDVLDHDSPPPGHVLCVKHVNGFTAAFSFALETQLTIGYGTMYPNADCPTAIALL 150
D. choprae         76 LLHWLLFAVLWYLLARVNGDLDVLDHDSPPPGHVLCVKHVNGFTAAFSFALETQLTIGYGTMYPNADCPTAIALL 150
D. erythromicron   76 LLHWLLFAVLWYLLARVNGDLDVLDHDSPPPGHVLCVKHVNGFTAAFSFALETQLTIGYGTMYPNADCPTAIALL 150
D. margaritatus    76 LLHWLLFAVLWYLLARVNGDLDVLDHDSPPPGHVLCVKHVNGFTAAFSFALETQLTIGYGTMYPNADCPTAIALL 150
cons.                 ***************************************************************************

                           dG→E                                     R/H      L/P
                             M2      rF→L                         G/S      N/T           A/S
D. rerio          151 ALQMLLGLMLEAFITGAFVAKFSRPQKRCDGILFSPQAVVCEQKGQRCLMFRVCNLQPQPLVDVSVSAVLYEERD 225
D. aesculapii     151 ALQMLLGLMLEAFITGAFVAKFSRPQKRCGGILFSPQAVVCEQKGQRCLMFRVCNLQPQPLVDVSVSAVLYEERD 225
D. kyathit        151 ALQMLLGLMLEAFITGAFVAKFSRPQKRCGGILFSPQAVVCEQKGQRCLMFRVCNLQPQPLVDVSVSAVLYEERD 225
D. nigrofasciatus 151 ALQMLLGLMLEAFITGAFVAKFSRPQKRCGGILFSPQAVVCEQKGQRCLMFRVCNLQPQPLVDVSVSAVLYEERD 225
D. tinwini        151 ALQMLLGLMLEAFITGAFVAKFSRPQKRCGGILFSPQAVVCEQKGQRCLMFRVCNLQPQTLVDVSVSAVLYEERD 225
D. albolineatus   151 ALQMLLGLMLEAFITGAFVAKFSRPQKRCGGILFSPQAVVCEQKGQRCLMFRVCNLQPQPLVDVSVSAVLYEERD 225
D. choprae        151 ALQMLLGLMLEAFITGAFVAKFSRPQKRCDGILFSPQAVVCEQKGQRCLMFRVCNLQPQPLVDVSVSAVLYEERE 255
D. erythromicron  151 ALQMLLGLMLEAFITGAFVAKFSRPQKRCGGILFSPQAVVCEQKGQRCLMFRVCNLQPQPLVDVSVSAVLYEERD 225
D. margaritatus   151 ALQMLLGLMLEAFITGAFVAKFSRPQKRCGGILFSPQAVVCEQKGQRCLMFRVCNLQPQPLVDVTVSAVLYEERD 225
cons.                 *****************************.*****************************.****:*********:

D. rerio          226 DHELHQTALEFSIDSLGSRSCPLFLSPLTFFHPLNPSTPFINNPSSQTHFELVVFLTATQESTGSGYHKRTSYLP 300
D. aesculapii     226 DHELHQTALEFSIDNLGSKSCPLFLSPLTFFHPLNPSTPFINNPSSQTHFELVVFLTATQESTGSGYHKRTSYLP 300
D. kyathit        226 DHELHQTALEFSIDNLGSRSCPLFLSPLTFFHPLNPSTPFINNPSSQTHFELVVFLTATQESTGSGYHKRTSYLP 300
D. nigrofasciatus 226 DHELHQTALEFSIDSLGSRSCPLFLSPLTFFHPLNPSTPFINNPSSQTHFELVVFLTATQESTGSGYHKRTSYLP 300
D. tinwini        226 DHELHQTALEFSIDNLGSRSCPLFLSPLTFFHPLNPSTPFINNPSSQTHFELVVFLTATQESTGSGYHKRTSYLP 300
D. albolineatus   226 DHELHQTALEFSTDNLGSRSCPLFLSPLTFFHPLSPSTPFINNPSSQTHFELVVFLTATQESTGSGYHKRTSYLP 300
D. choprae        226 DHELHQTALEFSIDNLGSRSCPLFLSPLTFFHPLNPSTPFINNPSSQTHFELVVFLTATQESTGSGYHKRTSYLP 300
D. erythromicron  226 DHELHQTALEFSIDNLGSRSCPLFLSPLTFFHPLNPSTPFINNPSSQTHFELVVFLTVTQESTGSGYHTRTSYLP 300
D. margaritatus   226 DHELHQTALEFSIDNLGSRSCPLFLSPLTFFHPLNPSTPFINNPSSQTHFELVVFLTVTQESTGSGYHMRTSYLP 300
cons.                 ************ *.***:***************.**********************.********** ******

                                 T/M        rY→X V/A
D. rerio          301 DEIQYGYCFSKVTSVHQNKTPNMRYFDTVPCPLTLANTHTTDTPDKEHVVVQLNGEGSDRVE 362
D. aesculapii     301 DEIQYGYCFSKVTSVHQNKTPNMRYFDTVPCPLTLANTHTTDTPDKEHVVVQLNGEGSDRVE 362
D. kyathit        301 DEIQYGYCFSKVTSVHQNKTPNMRYFDTVPCALTLANTHTTDTPDKEHVVVQLNGEGSDRVE 362
D. nigrofasciatus 301 DEIQYGYCFSKVTSVHQNKTPNMRYFDTVPCQLTLTNTHTTDTPDKEHVVVQLNGEGSDRVE 362
D. tinwini        301 DEIQYGYCFSKVTSVRQNKTPNMRYFDTVPCQLTLANTHTTDTPDKEHVVVQLNGEGSDRVE 362
D. albolineatus   301 DEIQYGYCFSKVASVHQNKTPNMRYFDTVPCPLTLANTHTTDTPDKEHVVVQLNGEGSDRVE 362
D. choprae        301 DEIQYGYCFSKVTSVHQNKTPNMRYFDTVPCPLTLANTHTTDTPDKEHVVVQLNGEGSDRVE 362
D. erythromicron  301 DEIQYGYCFSKVTSVHQNKTPNMRYFDTVPCPMALSNTHTTDMPDKEHVVVQLNGEGSDHVE 362
D. margaritatus   301 DEIQYGYCFSKVMAVHQNKTPNMRYFDTVPCPLALSNTHTTDTPDKEHVVVQLNGEGSDHVE 362
cons.                 ************ :*:*************** ::*:****** ****************:**
```

Supplementary Fig. 5: Sequence alignment of Kcnj13 orthologues from *Danio* species.

Kcnj13 sequences from *D. rerio*, *D. aesculapii*, *D. kyathit*, *D. nigrofasciatus*, *D. tinwini*, *D. albolineatus*, *D. choprae*, *D. margaritatus*, *D. erythromicron*. Amino acids evolved between *D. rerio* and *D. aesculapii* (magenta), *D. tinwini* (yellow) and *D. choprae* (cyan). Dominant (d) or recessive (r) mutations in *D. rerio* Kcnj13[1-5] (blue). Amino acid polymorphisms in *D. rerio* (dark grey). Transmembrane domains (M1/M2) (light grey blocks) and the P-loop (H5) (dark grey block).

Supplementary Table 1 | List of targeted genes.

target	CRISPR target sequence (5'-3')	genotyping
D. aesculapii csf1ra	GGCCTTTAACCTGGTCGGTC	T2143, T2144
D. aesculapii cx39.4	GGACTCACAGCCGGGCTGTT	T2145, T2146
D. aesculapii cx41.8	GAACTTTCTAGAAGAAGTCC	MP92, MP318
D. aesculapii igsf11	GCTGAAAGTACAGGGCAAGA	MP330, MP 331
D. aesculapii kcnj13	TGCTGTATTATGGTACCTGC	T963, T964
D. aesculapii mitfa	GGAGCGCTGGCTCCGGGTCC	T2147, T2148
D. aesculapii mpv17	GGTGCTTTTCTGGGAATAAC	T2149, T2150
D. rerio igsf11	GGACGCAATATAGGAGTGAT	T1449, T1450
D. rerio kcnj13 (1)	GGCAAGCAGCGCGATGGCAG	T2139, T2140
D. rerio kcnj13 (2)	GGCTGGCGCTACGGTGGCGG	T963, T964

Supplementary Table 2 | Primer pairs used for the generation of sgRNAs.

target	forward	reverse
D. aesculapii csf1ra	AAACGACCGACCAGGTTAAAGG	TAGGCCTTTAACCTGGTCGGTC
D. aesculapii cx39.4	AAACAACAGCCCGGCTGTGAGT	TAGGACTCACAGCCGGGCTGTT
D. aesculapii mitfa	AAACGGACCCGGAGCCAGCGCT	TAGGAGCGCTGGCTCCGGGTCC
D. aesculapii mpv17	AAACGTTATTCCCAGAAAAGCA	TAGGTGCTTTTCTGGGAATAAC
D. rerio csf1ra	AAACGACCGACCAGGTTAAAGG	TAGGCCTTTAACCTGGTCGGTC
D. rerio igsf11	AAACATCACTCCTATATTGCGT	TAGGACGCAATATAGGAGTGAT
D. rerio kcnj13 (1)	AAACCCGCCACCGTAGCGCCAG	TAGGCTGGCGCTACGGTGGCGG
D. rerio kcnj13 (2)	AAACCTGCCATCGCGCTGCTTG	TAGGCAAGCAGCGCGATGGCAG

Supplementary Table 3 | Primers used for genotyping.

primer name	sequence (5'-3')
MP318	AGCTGTGCCCAGAACCAAGA
MP330	CCCCCATGCATTTTATTTGACCA
MP331	CTGAATTCAGAAAGGAGGAGGT
MP92	CTCCCTTCCATTCACACTACC
T963	GAAACTATTCTTGCCGTGACTTG
T964	TCAAACAAACCTGGGTGTGGAC
T1449	TCATCTACCAGAGTGGTCAG
T1450	CCTAAACTTTTGCAGCACAG
T2139	TCAATGGAGACCTGGATGTC
T2140	TGGACCAAAGTGTGAAAGC
T2143	TGCCTGTGTTTATGTGTCG
T2144	AATGACCAAGAAGGATGAGC
T2145	GCCTCTAGGAACATGATTGG
T2146	GCTTCTCATTTCTAGCCCTC
T2147	GGCAACATTGGCGTTATCTC
T2148	TCTCACAGCATTCTGGCAC
T2149	CTGCCGTTTATATCTCCACAG
T2150	GGCTGAAAATTGGCTGATTG

Supplementary Table 4 | List of generated mutants.

mutant	mutation	description according to[13]
D. aesculapii Csf1ra^{t31ui}	4 bp deletion	recessive,<br>c.1500_1503delGGTC<br>p.Gly502LysfsX35
D. aesculapii Cx39.4^{t15ui}	8 bp deletion	recessive,<br>c.175_182delAAACAGCC<br>p.Lys59_ArgfsX2
D. aesculapii Cx39.4^{t59ui}	21 bp deletion	recessive,<br>c.168_188delCAACACCAAACAGCCCGGCTG<br>p.Asn57_Cys63del
D. aesculapii Cx41.8^{t18mp}	18 bp deletion	recessive,<br>c.41_58delTCCAGGAGCATTCAACCT<br>p.Val14AlaGlu15_Ser20del
D. aesculapii Igsf11^{t10mp}	1 bp insertion	recessive,<br>c.130dupT<br>p.Leu44PhefsX12
D. aesculapii Igsf11^{t19mp}	11 bp deletion	recessive,<br>c.129_139delCTTGCCCTGTA<br>p.Leu44PhefsX8
D. aesculapii Kcnj13^{t11mp}	4 bp deletion	recessive,<br>c.260_263delACCT<br>p.Tyr87CysfsX27
D. aesculapii Mitfa^{t30ui}	7 bp deletion	recessive,<br>c.194_200delGACCCGG<br>p.Gly65GlufsX22

D. aesculapii Mpv17^{t32ui}	4 bp deletion	recessive,<br>c.321_324delAATA<br>p.Ile108LeufsX6
D. rerio Igsf11^{t35ui}	17 bp deletion	recessive,<br>c.412_428delGTGATCGGCCTGACGGT<br>p.Ile138AlafsX19
D. rerio Kcnj13^{dt58ui}	6 bp deletion	dominant,<br>c.190_195delTGGCGG<br>p.Trp64Arg65del
D. rerio Kcnj13^{t24ui}	14 bp insertion	recessive,<br>c.436_437insGATGGAAGATGCTT<br>p.Ala146GlyfsX28

Supplementary References

1 Haffter, P. *et al.* Mutations affecting pigmentation and shape of the adult zebrafish. *Development genes and evolution* **206**, 260-276, doi:DOI 10.1007/s004270050051 (1996).

2 Iwashita, M. *et al.* Pigment pattern in jaguar/obelix zebrafish is caused by a Kir7.1 mutation: implications for the regulation of melanosome movement. *PLoS genetics* **2**, e197, doi:10.1371/journal.pgen.0020197 (2006).

3 Irion, U. *et al.* Gap junctions composed of connexins 41.8 and 39.4 are essential for colour pattern formation in zebrafish. *Elife* **3**, e05125, doi:10.7554/eLife.05125 (2014).

4 Henke, K. *et al.* Genetic Screen for Postembryonic Development in the Zebrafish (Danio rerio): Dominant Mutations Affecting Adult Form. *Genetics* **207**, 609-623, doi:10.1534/genetics.117.300187 (2017).

5 Silic, M. R. *et al.* Potassium Channel-Associated Bioelectricity of the Dermomyotome Determines Fin Patterning in Zebrafish. *Genetics* **215**, 1067-1084, doi:10.1534/genetics.120.303390 (2020).

6 Lee, M. M., Ritter, R., 3rd, Hirose, T., Vu, C. D. & Edwards, A. O. Snowflake vitreoretinal degeneration: follow-up of the original family. *Ophthalmology* **110**, 2418-2426, doi:10.1016/S0161-6420(03)00828-5 (2003).

7 Hejtmancik, J. F. *et al.* Mutations in KCNJ13 cause autosomal-dominant snowflake vitreoretinal degeneration. *Am J Hum Genet* **82**, 174-180, doi:10.1016/j.ajhg.2007.08.002 (2008).

8 Sergouniotis, P. I. *et al.* Recessive mutations in KCNJ13, encoding an inwardly rectifying potassium channel subunit, cause leber congenital amaurosis. *Am J Hum Genet* **89**, 183-190, doi:10.1016/j.ajhg.2011.06.002 (2011).

9 Khan, A. O., Bergmann, C., Neuhaus, C. & Bolz, H. J. A distinct vitreo-retinal dystrophy with early-onset cataract from recessive KCNJ13 mutations. *Ophthalmic Genet* **36**, 79-84, doi:10.3109/13816810.2014.985846 (2015).

10 Pattnaik, B. R. *et al.* A Novel KCNJ13 Nonsense Mutation and Loss of Kir7.1 Channel Function Causes Leber Congenital Amaurosis (LCA16). *Hum Mutat* **36**, 720-727, doi:10.1002/humu.22807 (2015).

11 Perez-Roustit, S. *et al.* Leber Congenital Amaurosis with Large Retinal Pigment Clumps Caused by Compound Heterozygous Mutations in Kcnj13. *Retin Cases Brief Rep* **11**, 221-226, doi:10.1097/ICB.0000000000000326 (2017).

12 Toms, M. *et al.* Missense variants in the conserved transmembrane M2 protein domain of KCNJ13 associated with retinovascular changes in humans and zebrafish. *Exp Eye Res* **189**, 107852, doi:10.1016/j.exer.2019.107852 (2019).

13 Ogino, S. *et al.* Standard mutation nomenclature in molecular diagnostics: practical and educational challenges. *J Mol Diagn* **9**, 1-6, doi:10.2353/jmoldx.2007.060081 (2007).
