# Supplementary material for: Evolution of the potassium channel gene Kcnj13 underlies colour pattern diversification in Danio fish
Source: Nat Commun. 2020 Dec 4;11:6230. doi: 10.1038/s41467-020-20021-6 (PMC7718271; doi:10.1038/s41467-020-20021-6)
Supplement: Supplementary file 3 — Reporting Summary [file 41467_2020_20021_MOESM3_ESM.pdf]

nature research

Corresponding author(s): Uwe Irion

Last updated by author(s): Nov 6, 2020

Reporting Summary

Nature Research wishes to improve the reproducibility of the work that we publish. This form provides structure for consistency and transparency in reporting. For further information on Nature Research policies, see our Editorial Policies and the Editorial Policy Checklist.

Statistics

For all statistical analyses, confirm that the following items are present in the figure legend, table legend, main text, or Methods section.

n/a	Confirmed	
[x]	[ ]	The exact sample size (*n*) for each experimental group/condition, given as a discrete number and unit of measurement
[x]	[ ]	A statement on whether measurements were taken from distinct samples or whether the same sample was measured repeatedly
[x]	[ ]	The statistical test(s) used AND whether they are one- or two-sided<br>*Only common tests should be described solely by name; describe more complex techniques in the Methods section.*
[x]	[ ]	A description of all covariates tested
[x]	[ ]	A description of any assumptions or corrections, such as tests of normality and adjustment for multiple comparisons
[x]	[ ]	A full description of the statistical parameters including central tendency (e.g. means) or other basic estimates (e.g. regression coefficient) AND variation (e.g. standard deviation) or associated estimates of uncertainty (e.g. confidence intervals)
[x]	[ ]	For null hypothesis testing, the test statistic (e.g. *F*, *t*, *r*) with confidence intervals, effect sizes, degrees of freedom and *P* value noted<br>*Give P values as exact values whenever suitable.*
[x]	[ ]	For Bayesian analysis, information on the choice of priors and Markov chain Monte Carlo settings
[x]	[ ]	For hierarchical and complex designs, identification of the appropriate level for tests and full reporting of outcomes
[x]	[ ]	Estimates of effect sizes (e.g. Cohen's *d*, Pearson's *r*), indicating how they were calculated

Our web collection on statistics for biologists contains articles on many of the points above.

Software and code

Policy information about availability of computer code

Data collection	RNA-Seq analysis was carried out using the Danio rerio GRCz11 genome build for all Danio species and STAR aligner with default settings. We found SNPs in the coding region of Kcnj13 and considered other resources, including the latest zebrafish reference genome assembly (GRCz11), the ENA deposition Zebrafish Genome Diversity (PRJEB20043, Wellcome Trust Sanger) and the Zebrafish Mutation Project.
Data analysis	The variant calling pipeline for all Danio species consisted of GATK 3.8 and 4 and picard from STAR-aligned bam files based on GATK Best-Practices pipeline. The full commands used can be found here: https://github.com/najasplus/STAR-deseq2 and https://github.com/najasplus/rnaseq_variant_calling. Variants were also called and checked using SAMtools, mpileup and bcftools. The protein sequence alignment was produced using T-coffee

For manuscripts utilizing custom algorithms or software that are central to the research but not yet described in published literature, software must be made available to editors and reviewers. We strongly encourage code deposition in a community repository (e.g. GitHub). See the Nature Research guidelines for submitting code & software for further information.

Data

Policy information about availability of data

All manuscripts must include a data availability statement. This statement should provide the following information, where applicable:

- Accession codes, unique identifiers, or web links for publicly available datasets
- A list of figures that have associated raw data
- A description of any restrictions on data availability

The data set (from RNA sequencing) generated during this study is available at The European Nucleotide Archive (ENA) accession number: PRJEB36360. (https://www.ebi.ac.uk/ena/browser/view/PRJEB36360)

Field-specific reporting

Please select the one below that is the best fit for your research. If you are not sure, read the appropriate sections before making your selection.

☐ Life sciences ☐ Behavioural & social sciences ☒ Ecological, evolutionary & environmental sciences

For a reference copy of the document with all sections, see nature.com/documents/nr-reporting-summary-flat.pdf

Ecological, evolutionary & environmental sciences study design

All studies must disclose on these points even when the disclosure is negative.

Study description	Danio rerio and Danio aesculapii mutants and Danio hybrids were phenotypically analyzed.
Research sample	Danio rerio, nine additional Danio species and Danio hybrids reared in the laboratory were used.
Sampling strategy	No statistical methods were used to predetermine sample size.
Data collection	Phenotypes were recorded by photography of the fish, genotypes were determined by sequence analysis.
Timing and spatial scale	Phenotypes were recorded from adult fish (>3 months).
Data exclusions	No data were excluded.
Reproducibility	All experiments were repeated at least once; in every experiment several (4 - 60) individuals were examined.
Randomization	The experiments were not randomized.
Blinding	The investigators were not blinded to allocation during experiments and outcome assessment.

Did the study involve field work? ☐ Yes ☒ No

Reporting for specific materials, systems and methods

We require information from authors about some types of materials, experimental systems and methods used in many studies. Here, indicate whether each material, system or method listed is relevant to your study. If you are not sure if a list item applies to your research, read the appropriate section before selecting a response.

Materials & experimental systems

n/a	Involved in the study	
☒	☐	Antibodies
☒	☐	Eukaryotic cell lines
☒	☐	Palaeontology and archaeology
☐	☒	Animals and other organisms
☒	☐	Human research participants
☒	☐	Clinical data
☒	☐	Dual use research of concern

Methods

n/a	Involved in the study	
☒	☐	ChIP-seq
☒	☐	Flow cytometry
☒	☐	MRI-based neuroimaging

Animals and other organisms

Policy information about studies involving animals; ARRIVE guidelines recommended for reporting animal research

Laboratory animals	Danio rerio, D. aesculapii, D. kyathit, D. nigrofasciatus, D. tinwini, D. albolineatus, D. choprae, D. erythromicron, D. margaritatus, D. dangila
Wild animals	No wild animals were used.
Field-collected samples	No samples were collected in the field.
Ethics oversight	All animal experiments were performed in accordance with the rules of the State of Baden-Württemberg, Germany, and approved by the Regierungspräsidium Tübingen.

Note that full information on the approval of the study protocol must also be provided in the manuscript.
